# Tunisian Table Olive Oil Traceability and Quality Using SNP Genotyping and Bioinformatics Tools

**DOI:** 10.1155/2019/8291341

**Published:** 2019-02-06

**Authors:** Rayda Ben Ayed, Ahmed Rebai

**Affiliations:** Molecular and Cellular Screening Processes Laboratory, Centre of Biotechnology of Sfax, BP 1177, 3018, University of Sfax, Tunisia

## Abstract

To enhance and highlight the authentication and traceability of table olive oil, we considered the analysis of 11 Tunisian table olive cultivars based on seven SNP molecular markers (SOD, CALC, FAD2.1, FAD2.3, PAL70, ANTHO3, and SAD.1) localized in six different genes. Accordingly, we assessed the potential genotype-phenotypes links between the seven SNPs, on the one hand, and the quantitative and qualitative parameters, on the other. The obtained genotypes were analyzed with computational biology tools based on bivariate analysis, multinomial logistic regression, and the Bayesian networks modeling. Obtained results showed that PAL70 SNP marker was negatively influenced by the phenol rate (*r* = -0.886;* p *** <**0.001), the oxidative stability (*r* = -0.884;* p *** <**0.001), traducing a direct effect of the PAL70 genotype deviations on the proportion of total phenol for each variety. Additionally, we revealed a significant association of SAD.1 marker with the content of the linolenic unsaturated fatty acids (C18:3;* p=0.046*). Moreover, SAD.1 was positively correlated with the saturated stearic acid C18:0 (*r* = 0.644;* p* = 0.032) based on multinomial logistic regression and Bayesian networks modeling, respectively. This research work provides better understanding and characterization of the quality of Tunisian table olive and supplies a significant knowledge and data information for table olive traceability and breeding.

## 1. Introduction

In the Mediterranean basin countries, olive is one of the most important agricultural products. It is used for olive oil extraction or processed as either table olives. These latter are chosen from cultivated olive trees (*Olea europaea* L.) with regard to their size, volume, taste, and other organoleptic properties that make them suitable for table consumption.

According to the current data provided by the International Olive Oil Council [1], the world production of table olive is evaluated at 2,953,500 tons in the 2017/2018 season showing an impressive increase of 211 % in the global production of table olives in the period between 1991 and 2018. The most dramatic raises have been noted in Egypt, Turkey, Spain, Algeria, Greece, Argentina, Iran, and Morocco.

Initially, table olive production was restricted to the producing regions, mainly European Union, Egypt, Turkey, Algeria, Morocco, Argentina, and Syria. However, nowadays, table olive production and exports have extended to other countries like USA and Jordan with 6 and 5 tons, respectively [1]. In recent years, several countries such as Tunisia, Argentina, Jordan, and Morocco have enhanced their production of table olives compared to the previous season unlike some producer countries that remained constant or sustained a cutback, like Syria by 47 % and Peru by 1 % [1].

According to our previous results from studies performed on main world table olive varieties [2], the genetic diversity and distribution of table olive varieties are related to several qualitative and quantitative parameters. Additionally, biological and organoleptic markers together with computational biology tools could help characteristics determination of table olives and hence start resolving its authenticity. Moreover, this study highlighted that some varieties could be more suitable as olive oil cultivars than table olive consumption regarding their high yield and consistent oil fruit content (22%) [2]. For these reasons, it is crucial to develop strategies and procedures of traceability and authentication that allows rapid and relevant identification and then valorisation of cultivars. Generally, traceability, authenticity, and detection of fraudulency in olive oil are performed by analytical techniques. However, biochemical approach and analyses are not sufficient to assess olive oil authenticity due to the influence of environmental conditions on oil components [3–5].

More recently, the use of DNA molecule-based analyses in olive oil becomes of a great interest to meet the needs of consumers and will be essential for studying the traceability of olive oil because of their several advantages, particularly, the reliability and reproducibility of results.

Seven SNPs localized in five different genes:* fatty acid desaturase, anthocyanidin synthase (ANS), calcium-binding protein, stearoyl-acyl carrier protein desaturase (SAD),* and* L-phenylalanine ammonia lyase*. The FAD2.1 and FAD2.3 SNPs are both harboured by the* FAD2* gene which is involved in the biosynthesis of highly unsaturated fatty acids (HUFA) from the precursor polyunsaturated fatty acids (PUFA) [6]. The third studied SNP, named ANTHO3, is localized in the* anthocyanidin synthase* gene, a 2-oxoglutarate iron-dependent oxygenase, and catalyzes the penultimate step in the biosynthesis of the anthocyanin class of flavonoids [7]. The CALC SNP is carried by the calcium-binding protein gene that is involved in response to abiotic constraints (salinity, cold, and drought) [8]. The fifth SNP localized in the* Stearoyl-acyl carrier protein desaturase* gene is involved in the desaturation of C18:0 to C18:1, monounsaturated oleic acid intermediates [9]. The sixth SNP, named SOD, is an insertion/deletion polymorphism type localized in Cu–Zn superoxide dismutase gene associated with the oxidative stress response [10, 11]. The last SNP is the PAL70, located in the* L-phenylalanine ammonia lyase* gene that is implicated in phenolic biosynthesis, including the formation of flavonoids, lignin, and hydroxycinnamic acids [12].

Our study aims to assess the correlations between the seven SNPs and table olive oils quality parameters and their efficiency in the authentication and traceability of Tunisian table olive oil.

## 2. Materials and Methods

### 2.1. Plant Material

Eleven Tunisian table olive cultivars were selected from north to south geographical regions of Tunisia (Chetoui, Tounsi, Meski, Oueslati, El Horr, Fakhari, Zarrazi, Chemchali, Besbessi, Fougi, and Toffehi). Two trees were sampled for each cultivar, and olive oil was extracted from each sample, followed by DNA extraction [13].

### 2.2. Olive Oil Extraction

Fully ripened fruits coming from different dual purpose and table Tunisian olive varieties served for olive oil extraction. Olive fruit samples were immediately after harvest carried and stored into the laboratory for further oil extraction. In order to obtain olive oil, 2.5 kg of stoned olives was grinded, and olive oil was extracted by mechanical press. Standard methods commonly used in oil factories were followed in the procedure of monovarietal oil extraction and obtention, including milling, mixing at 25°C for 30 min, and centrifugation for 3 min at 2000g and the final step for olive oil obtention was by natural decantation. Samples were stored at 4°C into dark glass bottles until analysis.

### 2.3. DNA Isolation

DNA extraction from olive oil was performed by using the QIAmp DNA tool mini kit (Qiagen) according to the protocol described by Ben Ayed et al. (2012) [12] with slight modifications. DNA quantification was carried out by spectrophotometry (Tecan GENIOS Plus spectrofluorometer) and with Hoechst H33258 dye incorporation. Dilution series of Lamda DNA (D150A Promega) were used with standard calibration. Finally, genomic DNA was diluted in TE buffer (10 mM Tris–HCl pH 8.1 mM EDTA pH 8) and stored at -20°C.

### 2.4. SNP Genotyping

We considered seven SNPs (FAD2.1, FAD2.3, ANTHO3, CALC, ACP1, SOD, and PAL70) in our study; all SNPs were selected in the coding regions of* FAD2, ANTHO, CALC, SAD1, SOD, and PAL70* genes, all of them being involved in fruits pomology and associated with olive oil composition and therefore easily correlatable to phenotypic characters.

The SNP SOD (insertion/deletion type) was genotyped by a simple polymerase chain reaction followed by revelation through agarose gel electrophoresis, whereas the other six SNPs (FAD2.1, FAD2.3, ANTHO3, CALC, ACP1, and PAL70) were genotyped by a polymerase chain reaction-restriction fragment length polymorphism (PCR-RFLP) method (Table 1). The PCR product (171 bp) of the SNP (ANTHO3) was digested by* MspI* restriction enzyme (Fermentas, LIFE SCIENCES) at 37°C overnight. This restriction enzyme recognizes the sequence AA/GG. The G-allele carrying PCR product was cleaved once by the enzyme generating two fragments (64 and 107 bp). The PCR product (476 bp) of SNP (CALC) was digested by* BstZI *restriction enzyme (Promega) at 50°C overnight. This restriction enzyme recognizes the sequence CC/GG. The C-allele carrying PCR product was cleaved once by the enzyme producing two fragments (316-160 bp). The two other SNPs (FAD2.1 and FAD2.3) were analyzed using PCR-RFLP. The PCR product 241bp of the SNP FAD2.1 and 240 bp of the SNP FAD2.3 were digested by* BamHI *restriction enzyme (Fermentas, Life Sciences) and* Alw26I*, respectively, at 37°C overnight. The sizes of the restriction fragments of PCR product were 224 and 17 bp and 130 and 110 bp for CC genotype of FAD2.1 SNP and FAD2.3 SNP, respectively. The PCR product (330 bp) of SNP (SAD.1) was digested by* TaqI *restriction enzyme (Vivantis) at 65°C for 16 hours. This restriction enzyme recognizes the sequence CC/TT. The C-allele carrying PCR product was cleaved twice by the enzyme producing four fragments (263, 158, 105, and 67 bp). The PCR product (400 bp) of the SNP (PAL70) was digested by* HinfI *restriction enzyme (Fermentas, Life Sciences). The size of the restriction fragments of PCR product was 308, 52, and 40 bp for AA genotype of PAL70 SNP.

All digestion products were separated by electrophoresis on 3% Nusieve ethidium bromide-stained agarose gels and visualized under UV light.

### 2.5. Statistical Analysis

The analysis of the correlation between SNP markers and the studied parameters was performed in different steps including numerous statistical methods. In the beginning, the Chi-square test was used to evaluate the differences between the classes of qualitative traits in allele and genotype frequencies. Subsequently, a student test was used for quantitative traits, to assess the significant difference between the means of genotype groups for each SNP. R software packages were used to study the association between SNP markers and quantitative and qualitative parameters. All tests were declared statistically significant when* P values* are <0.05. Thereafter, to study the relationship of the studied seven SNPs with quantitative traits, a variance multiway analysis was carried out. In addition, multinomial logistic regression was applied in order to test the associations of the seven SNPs with qualitative traits independently.

To draw the directed acyclic graph (DAG), we used the R language and the ‘grow shrink' algorithm. The algorithm proficiently filters links out of a full skeletal DAG, in which all nodes are primarily connected (excluding those having no relationships with others), based on tests of conditional independence between a pair of nodes given all possible subsets of the rest. Logical rules are applied to create the direction of links (conditional dependence between variables), so that cycles are not introduced and patterns of conditional independence are found in the data match the generated DAG. We predicted association power in the final DAG by calculating approximately the beta-coefficient for a regression for each potential causal effect in which the variable at the base of the arrow (‘cause') was considered a covariate, and the variable at the head of the arrow (‘effect') was considered the outcome or dependent variable [14].

## 3. Results and Discussion

### 3.1. Genotyping and Characteristics of the SNP Markers

The observed heterozygosity for the studied SNP markers ranged from 0.363 (SAD.1) to 0.909 (FAD2.1) (0.545 average), whereas the expected heterozygosity ranged from 0.297 to 0.495 with an average of 0.492 indicating a high level of heterozygosity for all markers (Table 1).

Polymorphism Information Content (PIC) value was determined for the studied table olive cultivars.

Table 1 shows that SOD Indel (PIC=0.595) and PAL70 SNP (0.496) markers have higher PIC values than the other studied markers, meaning that SOD and PAL70 are the most informative markers and therefore able to distinguish between our table olive cultivars. This result, together with mean value observed across the loci and table olive cultivars in the present study, is in accordance with the results previously obtained by Ben Ayed et al. (2014) [15]. SOD gene, for superoxide dismutase, encodes for an antioxidant enzyme that plays a pivotal role in protecting cells against superoxide radicals accumulation [16]. Phenylalanine ammonia lyase PAL70 catalyzes the reaction of trans-cinnamic acid formation* via *L-phenylalanine deamination and is therefore associated with phenolic compounds content in olives [17].

The highest discriminating power (DP) value 0.528 was shown for FAD2.3 marker. The mean value is 0.464. Using SSR markers, Reale et al. (2006) [18] and Muzzalupo et al. (2009) [19] obtained similar values, respectively, with DNA samples extracted from 65 olive cultivars and 39 Italian cultivars (0.38). However, the average values are lower than those found by Cipriani et al. (2002) [20] in 12 Italian cultivars (0.44) using SSR markers (0.71).* Fatty acid desaturase 2* gene which is involved in the biosynthesis of HUFA from PUFA precursor has been demonstrated to be associated with oleic/linoleic acid ratio content of olive oils from Tunisian olive oil cultivars [21].

The allele frequencies of the seven studied SNPs revealed a dominance of all markers, except for PAL70 whose two alleles displayed similar frequencies.

### 3.2. Association between SNP Polymorphisms and Olive Oil Quality Parameters

In order to illustrate the association between quality of the table olive cultivars and gene information, we applied the likelihood ratio test (LRT). Thus, a genome-wide association was carried out to identify table olive fruit quality susceptibility alleles. We studied 7 SNPs located in 6 genes for 11 table olive samples. Then, we evaluated the *p* values of the LRT and Ki_2_ tests. The results are summarized in Table 2 and demonstrated the absence of any significant associations between the seven SNPs (SOD, FAD2.1, FAD2.3, ANTHO3, CALC, SAD.1, and PAL70) genotypes and none of the qualitative traits is considered in this work.

As shown in Table 3, significant associations between CALC SNP and one parameter which is palmitic acid (C16:0) were found. However, the average rate of C16:0 between the heterozygote varieties with CG-CALC and GG-CALC genotypes (*p = 0.04*) was significantly different. This positive association among CALC polymorphisms and C16:0 parameter suggests that the heterozygote varieties with CG genotypes produce, on average, higher levels of C16:0 than GG genotypes varieties. As shown in Table 5, this significant correlation is proved by multinomial logistic regression modeling (*p* = 0.039).

Regarding the FAD2.1 SNP, a highly significant association with *β*-sitosterol (*p=0.018*) quantitative parameter is proved. Similar result is generated by multinomial logistic regression (*p*=0.022) (Table 5).

Besides, FAD2.3 SNP was found to be highly associated with three quantitative parameters, namely, acidity (*p*=0.024), rate of carotene (*p=0.013*), and cholesterol content (*p=0.048*) (Table 3). The homozygous varieties (CC- FAD2.3) were the main genotypes concerned by these positive associations. FAD gene is known to be involved in the synthesis pathway of the unsaturated fatty acid [6], suggesting a direct effect of FAD2.3 genotypic variations on the rate of PUFA (such as C18:2 and C18:3) for each variety and hence influenced the acidity parameter. In fact, the homozygous varieties CC (Toffehi, Fakhari, and Fougi cultivars) have a higher acidity than the heterozygous varieties CG (other cultivars). Nevertheless, the cholesterol rate was significantly higher in the varieties carrying the homozygous genotype CC-FAD2.3 (particularly Toffehi with a cholesterol rate of 1.98, and Fakhari with 2.17), than the heterozygous genotypes (Table 3). Moreover, CC varieties contain more carotene pigment than the heterozygous genotypes. Moreover, the FAD2.3 SNP marker is significantly associated with the acidity and the cholesterol rate by using the analysis of variance (*p*=0.006,* p<0.001*) (Table 4) and the multinomial logistic regression modeling (*p*=0.026,* p<0.001*) (Table 5), respectively.

The study of ANTHO3 SNP led to the identification of two genotypes: AA and AG, with a level of AG-ANTHO3 heterozygosity of 63.63 %. A significant association was established with the rate of total sterols (*p = 0.042*), which has a higher average for heterozygous cultivars (AG) (representing 63.63%  % of all samples) (Table 3). This significant correlation is proved by multinomial logistic regression modeling (*p* = 0.04) (Table 5).

Moreover, PAL70 SNP is clearly associated with 3 parameters, namely, chlorophyll, total phenol contents, and the oxidative stability (Table 3). However, a variability of the rate of chlorophyll pigment among the heterozygote varieties with AG-PAL70 and AA-PAL70 genotypes (*p=0.002*) was noted. A positive correlation between the total phenol content and the genotype variation for this marker (*p<0.001*) could also be observed, where the varieties with AA genotypes displayed the highest total phenolic content. Regarding the relationship with oxidative stability (*p<0.001*), the homozygous varieties AA behaved with better oil stability than the heterozygous varieties AG. Moreover, the PAL70 SNP marker is significantly associated with the chlorophyll rate (*p=0.031*) by using the analysis of variance (Table 4).

Additionally, multivariate analyses were used to study the association between olive oil parameters and the PAL70 SNP marker, showing an important significant association between this SNP marker and the acidity parameter (Tables 4 and 5). The *p* values of this association were *p*=0.004 and *p* = 0.036 using the analysis of variance and the multinomial logistic regression modeling, respectively.

The association between total phenol rate and the PAL70 SNP marker is biologically relevant since the PAL70 marker is located in the* L-phenylalanine ammonia lyase* gene that is involved in the biosynthesis of phenylpropanoid compounds [12].

**Figure 1:**
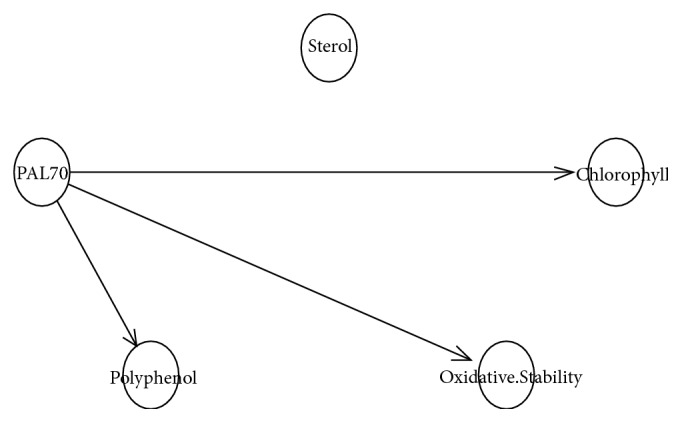
Directed acyclic graph representing possible PAL70 SNP marker connections with total phenol, oxidative stability, and chlorophyll.

The relationship between the PAL70 SNP and the phenol level was assessed by Bayesian networks modeling. The derived DAG (directed acyclic graph) is shown in Figure 1 where directed arrows indicate the direction of ‘causal' influence between variables. Three direct influences are identified: effect of PAL70 marker on the phenol rate, oxidative stability, and chlorophyll content. In fact, Figure 1 shows that the PAL70 SNP was negatively influenced by the phenol rate (*r*=-0.886;* p ***<**0.001), the oxidative stability (*r*=-0.884;* p ***<**0.001), and the chlorophyll (*r*=-0.814;* p*=0.002). Furthermore, PAL70 node was not influenced by the sterol level (*r*=0.223;* p*=0.510).

The oxidative stability of the olive oil samples is directly influenced by the total phenol level. Besides, total phenol amount is directly influenced by the PAL70 marker. The latter plays a key role in the total phenol level of each of the olive oil varieties. This finding could be explained by the fact that PAL70 SNP is located within a gene involved in the phenolic biosynthesis (Balsa et al. 1979), suggesting the direct effect of the PAL70 genotype variations on the percentage of total phenol for each variety.

For SAD.1 SNP study, two genotypes were identified: TT and CT. About 64 % of the varieties were homozygous TT-SAD.1 and including both two dual-use cultivars (Chemchali and Oueslati) and two table olive cultivars (Toffehi and Fakhari). Two significant associations of this marker were shown with the accumulation of the linolenic unsaturated fatty acids (C18:3;* p=0.046*) and with the rate of carotene (*p=0.005*) (Table 3).

The two significant correlations between SAD.1 SNP marker and the content of carotene pigments (*p*=0.01) and C18:3 (*p*=0.043) are confirmed by multinomial logistic regression modeling (Table 5).

The relationship between the molecular marker SAD.1 and fatty acid composition was also analyzed by Bayesian networks modeling.

**Figure 2:**
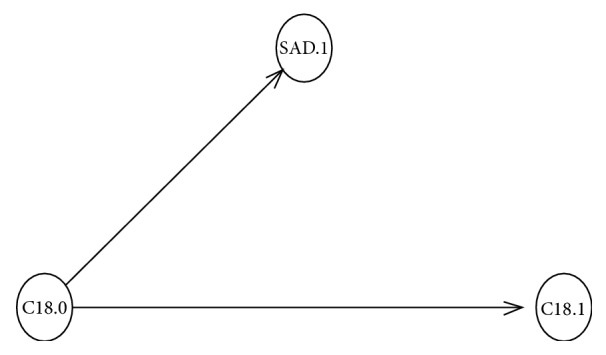
Directed acyclic graph representing possible SAD.1 SNP marker connections with stearic acid C18:0 and oleic acid C18:1.

Firstly, 3 nodes were considered as represented in Figure 2. Pearson correlation coefficients among fatty acid compositions in olive oil varieties are presented in Table 6. Moreover, SAD.1 was positively influenced by the saturated stearic acid C18:0 (*r* = 0.644;* p* = 0.032).

SAD gene is known to be associated with the transformation of the saturated stearic fatty acid C18:0 to the monounsaturated oleic fatty acid C18:1, therefore, suggesting the direct effect of the SAD.1 genotype variations on the fatty acid content [6].

## 4. Conclusions

SNP genotyping is a valuable approach for marker assisted selection in crops. For this reason, we studied in this current work the correlations between the six SNPs and table olive oils quality parameters and their usefulness in the traceability of Tunisian table olive oil. We revealed that PAL70 SNP marker was negatively influenced by the phenol rate (*r*=-0.886;* p ***<**0.001) and the oxidative stability (*r*=-0.884;* p ***<**0.001). Besides, we reported a significant association of SAD.1 marker with the accumulation of the linolenic unsaturated fatty acid (C18:3;* p=0.046*) and that SAD.1 was positively influenced by the saturated stearic acid C18:0 (*r*=0.644;* p*=0.032) based on multinomial logistic regression and Bayesian networks modeling, respectively. To the best of our knowledge, this is the first work that analyses the SNP markers of Tunisian table olive oil and the quality of the oil.

## Figures and Tables

**Table 1:** Characteristics of SNP studied markers.

**Gene name**	**GenBank Accession Number**	**SNP code**	**Tm** ^**a**^	**H0**	**He**	**P** **I** **C** ^**α**^
*Phenylalanine ammonia lyase*	AY738639	PAL70 (A/G)	60	0.545	0.396	**0.496**
*Calcium Binding Protein*	AF078680	SNP-H(C/G)	60	0.727	0.462	0.396
*Anthocyanidin synthase*	AF384050	SNP-I(G/A)	57	0.636	0.433	0.463
*Fatty acid desaturase*	AY083163	FAD2.1(T/C)	57	**0.909**	**0.495**	0.165
		FAD2.3(C/G)		0.636	0.433	0.463
*Stearoyl-ACP desaturase*	U58141	SAD.1(A/G)	57	0.363	0.297	0.463
*Cu-Zn-superoxide dismutase*	AF426829	SOD (InDel)	57	0.545	**0.495**	**0.595**

Mean				0,545	0.492	0.488

a: annealing temperature for PCR amplification.

*α*: for each locus the polymorphism content information (PIC).

**Table 2:** Association between ANTHO3 and PAL70 genotypes and qualitative parameters.

**Polymorphisms**		**ANTHO3**			**PAL70**		

**Qualitative parameters**		**GA**	**AA**	**Chi-square**	**AA**	**AG**	**Chi-square**
		**(**%**)**	**(**%**)**	LRT^∗^	**(**%**)**	**(**%**)**	RLT^∗^

**Fruit Form **	*Ovoid*	1.9	1.1	3.592	1.1	1.9	1.946
	*Elongate*	4.5	2.5	0,166^∗^	2.5	4.5	0.378^∗^
	*Spherical*	0.6	0.4		0.4	0.6	

**Stone Form**	*Elongate*	4.5	2.5	5.135	3.2	3.8	3.221
	*elliptic*	1.3	0.7	0.052^∗^	0.9	1.1	0.2^∗^
	*ovoid*	1.3	0.7		0.9	1.1	

**Symmetry stone **	*Asymmetric*	3.8	2.2	5.238	2.7	3.3	7.639
	*Slightly asymmetric*	1.9	1.1	0,155^∗^	1.4	1.6	0.054^∗^
	*Symmetric*	0.6	0.4		0.5	0.5	
	*Slightly symmetric*	0.6	0.4		0.5	0.5	

Bold values: each variable that has statistical significance for all tests was declared when *P* values are <0.05.

**(a):** 

Parameters		Cholesterol		*β*-sitosterol		Chlorophyll		Carotene		FWM	
SNPs		Mean ±SD	*P*	Mean ±SD	*P*	Mean ±SD	*P*	Mean ±SD	*P*	Mean ±SD	*P*
**CALC**	**CG**	1.52 ±0.34	*0.405(0.224)*	1387.13 ±221.97	*0.315(0.47)*	1.71 ±1.17	*0.75(0.73)*	4.64 ±2.95	*0.7(0.52)*	3.58 ±2.92	*0.641(0.663)*
	**GG**	1.34 ±0.12		1573.67 ±360		1.46 ±0.94		3.93 ±0.11		4.55±3.01	
**FAD 2.1**	**CT**	1.48 ±0.32	*0.908*	1383 ±198	***0.018***	1.77 ±1.04	*0.244*	4.49 ±2.62	*0.86*	3.43 ±2.6	*0.13*
	**TT**	1.44 ±0.32		1981 ±198		0.4 ±1.04		4 ±2.62		8 ±02.6	
**FAD 2.3**	**CC**	1.71 ±0.043	*0.048(0.186)*	1423 ±312	*0.89(0.9)*	1.75 ±0. 78	*0.82(0.79)*	6.7 ±2.58	***0.013(0.064)***	2.6 ±0.62	*0.29(0.18)*
	**CG**	1.34 ±0.078		1446 ±253		1.58 ±1.26		3.16 ±1.30		4.56 ±3.39	
**ANTHO3**	**GA**	1.56 ±0.36	*0.224(0.128)*	1539 ±261	*0.087(0.053)*	1.2 ±0.806	*0.064(0.118)*	4.54 ±3.1	*0.87(0.84)*	4.85 ±3.08	*0.123(0.063)*
	**AA**	1.32 ±0.047		1260 ±152		2.42 ±1.13		4.28 ±1.17		2.08 ±1.06	
**PAL70**	**AA**	1.31 ±0.07	*0.115(0.109)*	1342 ±92.266	*0.287(0.264)*	2.56 ±0.68	***0.002(0.003)***	4.2 ±1.03	*0.78(0.76)*	2.1 ±0.84	*0.055(0.054)*
	**AG**	1.61 ±0.367		1518 ±334.7		0.88 ±0.63		4.65 ±3.38		5.3 ±3.1	
**SAD.1**	**TT**	1.345 ±0.07	*0.055(0.198)*	1424 ±281	*0.828(0.825)*	1.2 ±0.91	*0.064(0.082)*	3.06 ±1.2	***0.005(0.039***)	4.85 ±3.16	*0.123(0.06)*
	**CT**	1.7 ±0.436		1462 ±258		2.425 ±0.95		6.88 ±2.34		2.08 ±0.45	

**(b):** 

Parameters		C16:0		C16:1		C18:0		C18:3		I/S	
SNPs		Mean ±SD	*P*	Mean ±SD	*P*	Mean ±SD	*P*	Mean ±SD	*P*	Mean ±SD	*P*
**CALC**	**CG**	15.08 ±2.1	***0.04(0.041)***	1.53 ±0.54	*0.057 * ***(0.025)***	1.9 ±0.75	*0.686(0.523)*	14.65 ±5.1	*0.76(0.626)*	4.95 ±0.91	*0.075 * ***(0.027)***
	**GG**	11.83 ±1.58		0.8 ±0.30		2.08 ±0.17		12.53 ±4.7		6.08 ±0.45	
**FAD 2.1**	**CT**	14.33 ±2.51	*0.562*	1.36 ±0.61	*0.694*	1.93 ±0.67	*0.815*	0.63 ±0.15	*0.83*	5.21 ±0.98	*0.615*
	**TT**	12.75 ±2.51		1.1 ±0.61		2.1 ±0.67		0.6 ±0.15		5.75 ±0.98	
**FAD 2.3**	**CC**	15.06 ±0.6	*0.39(0.27)*	1.73 ±0.32	*0.084 * ***(0.049)***	2.25 ±0.92	*0.254(0.387)*	0.57 ±0.13	*0.337(0.32)*	4.7 ±0.43	*0.206(0.127)*
	**CG**	13.69 ±2.97		1.1 ±0.58		1.77 ±0.39		0.66 ±0.14		5.54 ±1.07	
**ANTHO3**	**GA**	14.23 ±2.62	*0.95(0.94)*	1.15 ±0.56	*0.19(0.19)*	2.11 ±0.7	*0.26(0.22)*	0.66 ±0.15	*0.54(0.45)*	5.14 ±0.96	*0.61(0.63)*
	**AA**	14.13 ±2.42		1.65 ±0.53		1.65 ±0.47		0.57 ±0.13		5.46 ±1.03	
**PAL70**	**AA**	12.85 ±2.63	*0.095(0.118)*	1.21 ±0.68	*0.542(0.556)*	1.88 ±0.42	*0.775(0.763)*	0.55 ±0.07	*0.108(0.097)*	5.85 ±0.97	*0.054(0.073)*
	**AG**	15.31 ±1.72		1.44 ±0.52		2. ±0.81		0.69 ±0.16		4.76 ±0.63	
**SAD.1**	**TT**	14.34 ±2.64	*0.809(0.804)*	1.25 ±0.63	*0.546(0.528)*	1.67 ±0.41	*0.063(0.148)*	0.695 ±0.14	*0.046(0.019)*	5.271 ±0.86	*0.958(0.963)*
	**CT**	13.94 ±2.36		1.48 ±0.53		2.41 ±0.75		0.521±0.048		5.238±1.23	

Bold values: each variable that has statistical significance for all tests was declared when *P*-values are <0.05.

***P*:** *P*-value of student test;** SD**: standard deviation; **FWM**: Fruit Weight at Maturation.

**(c):** 

Parameters		MG/fruit		Polyphenol		Total Sterols		Acidity	
SNPs		Mean ±SD	*P*	Mean ±SD	*P*	Mean ±SD	*P*	Mean ±SD	*P*
**CALC**	**CG**	0.74 ±0.55	*0.54(0.56)*	165 ±66	*0.21(0.442)*	1682±315	*0.26(0.35)*	0.29 ±0.058	*0.308(0.191)*
	**GG**	0.98 ±0.54		249 ±151		1946 ±374		0.25 ±0.03	
**FAD 2.1**	**CT**	0.72 ±0.49	*0.125*	199 ±93.23	*0.233*	1715 ±327	*0.245*	0.28 ±0.05	*0.974*
	**TT**	1.6 ±0.49		74.4 ±93.23		2142 ±327		0.28 ±0.05	
**FAD 2.3**	**CC**	0.55 ±0.02	*0.255(0.151)*	163.28 ±70.54	*0.54(0.5)*	1739 ±428	*0.91(0.92)*	0.32 ±0.06	***0.024(0.1)***
	**CG**	0.95 ±0.63		202.2 ±110.8		1763 ±309		0.25 ±0.02	
**ANTHO3**	**GA**	1.02 ±0.56	*0.075 * ***(0.033)***	169.64 ±116	*0.43(0.32)*	1904 ±316	***0.042(0.022)***	0.28 ±0.06	*0.61(0.51)*
	**AA**	0.43 ±0.13		220 ±39.08		1492 ±176		0.27 ±0.01	
**PAL70**	**AA**	0.5 ±0.19	*0.085(0.079)*	277 ±57.54	***0(0.001)***	1676 ±298	*0.51(0.5)*	0.25 ±0.02	*0.123(0.112)*
	**AG**	1.06 ±0.61		113.92 ±36.47		1819 ±378		0.3 ±0.06	
**SAD.1**	**TT**	0.97 ±0.51	*0.18(0.097)*	191.2 ±109.53	*0.894(0.885)*	1741.9±340	*0.878(0.883)*	0.26±10.03	*0.098(0.223)*
	**CT**	0.51 ±0.09		182.53 ±82.17		1777 ±380		0.317 ±0.07	

**(d):** 

Parameters		Δ-5-avenasterol		Oxidative stability		campesterol	
SNPs		Mean ±SD	*P*	Mean ±SD	*P*	Mean ±SD	*P*
**CALC**	**CG**	194 ±83.93	*0.234(0.261)*	39.63 ±17.17	*0.32(0.42)*	54.07 ±15.71	*0.78(0.78)*
	**GG**	122.6 ±78.9		52.67 ±21.93		51.04 ±15.4	
**FAD 2.1**	**CT**	188 ±74.9	*0.077*	44.7 ±18.69	*0.41*	51.77 ±14.84	*0.323*
	**TT**	31.8 ±74.9		28 ±18.69		68.04 ±14.84	
**FAD 2.3**	**CC**	205.75 ±88.8	*0.38(0.4)*	40.75 ±11.14	*0.76(0.71)*	58.72 ±15.57	*0.38(0.4)*
	**CG**	156.8 ±84.84		44.57 ±22.31		50.12 ±14.76	
**ANTHO3**	**GA**	188 ±105	*0.51(0.4)*	37.29 ±20.64	*0.17(0.097)*	58.56 ±16.65	*0.12( * ***0.06)***
	**AA**	150 ±26		53.5 ±7.5		43.95 ±2.28	
**PAL70**	**AA**	162.2 ±20.62	*0.68(0.66)*	60.2 ±6.14	*0.000*	43.4 ±3.85	***0.037(0.039)***
	**AG**	184.97 ±118.17		29 ±10.88		61.45 ±15.96	
**SAD.1**	**TT**	156.9 ±84.78	*0.393(0.41)*	42.29 ±21.39	*0.844(0.826)*	50.1 ±14.77	*0.381(0.398)*
	**TC**	205.5 ±89.1		44.75 ±14.56		58.77 ±15.51	

**Table 4:** *P values* of Fisher tests for the association study between SNP markers and the oil quality characteristics.

**Model**							
	**SOD**	**CALC**	**ANTHO3**	**FAD2.1**	**FAD2.3**	**PAL70**	**SAD.1**

**FWM**	-* *-	0.894	**0.022**	-* *-	**0.008**	**0.017**	**0.012**
**MG/fruit**	-* *-	0.955	**0.028**	-* *-	**0.009**	**0.027**	**0.018**
**acidity**	-* *-	0.341	0.082	-* *-	**0.006**	**0.004**	**0.001**
**C16:0**	-* *-	0.877	0.782	-* *-	**0.011**	0.844	0.606
**C16:1**	-* *-	0.088	0.783	-* *-	0.215	0.640	0.351
**C18:0**	-* *-	0.227	0.853	-* *-	0.175	0.140	0.205
**C18:1**	-* *-	0.799	0.562	-* *-	0.059	0.364	0.294
**C18:2**	-* *-	0.963	0.336	-* *-	0.182	0.289	0.185
**C18:3**	-* *-	**0.033**	0.651	-* *-	0.687	**0.026**	0.058
**C20:0**	-* *-	0.107	0.910	-* *-	0.079	0.149	0.174
**I/S**	-* *-	0.557	0.899	-* *-	0.066	0.794	0.592
**Polyphenol**	-* *-	**0.035**	0.048	-* *-	0.18	0.895	0.487
**Oxidative Stability**	-* *-	0.696	0.054	-* *-	**0.012**	0.935	0.229
**chlorophyll**	-* *-	0.625	0.832	-* *-	0.168	**0.031**	0.9
**carotene**	-* *-	0.065	0.169	-* *-	0.188	0.065	0.195
**β** **-sitosterol**	-* *-	0.275	0.273	-* *-	0.503	0.632	0.880
**Campesterol**	-* *-	0.458	0	-* *-	0.702	0.56	0.715
Δ**-5-avenasterol**	-* *-	0.906	0.136	-* *-	0.844	0.991	0.838
**cholesterol**	-* *-	0.125	**0.018**	-* *-	**0**	**0**	**0**
**Total Sterols**	-* *-	0.694	0.182	-* *-	0.564	0.92	0.956
**(Uvaol+erythro)/total sterol**	-* *-	0.744	0.217	-* *-	0.172	0.292	0.095

Bold values: each variable that has statistical significance for all tests was declared when *P values* are < 0.05; **∗**: each variable that has statistical significance for all tests was declared when *P values* are <0.05 and has biological relevance.

**Table 5:** *P* values given by the binary logistic regression analysis.

**Model**							
	**SOD**	**CALC**	**ANTHO3**	**FAD2.1**	**FAD2.3**	**PAL70**	**SAD.1**

**FWM**	-* *-	0.599	0.102	0.107	0.246	0.134	0.102
**MG/fruit**	-* *-	0.493	0.064	0.103	0.213	0.183	0.149
**acidity**	-* *-	0.261	0.565	0.970	**0.026**	0.036	0.082
**C16:0**	-* *-	**0.039**	0.943	0.514	0.345	0.682	0.784
**C16:1**	-* *-	0.051	0.159	0.657	0.071	0.462	0.497
**C18:0**	-* *-	0.647	0.226	0.791	0.212	0.231	0.055
**C18:1**	-* *-	0.146	0.519	0.173	0.710	0.351	0.607
**C18:2**	-* *-	0.501	0.497	0.144	0.778	0.261	0.451
**C18:3**	-* *-	0.728	0.321	0.808	0.288	0.147	**0.043**
**C20:0**	-* *-	0.889	0.629	0.914	0.138	0.317	0.246
**I/S**	-* *-	0.065	0.574	0.570	0.170	0.870	0.952
**polyphenol**	-* *-	0.176	0.378	0.194	0.498	0.795	0.880
**oxidative Stability**	-* *-	0.273	0.141	0.365	0.729	0.407	0.823
**chlorophyll**	-* *-	0.723	0.057	0.203	0.798	0.420	0.057
**carotene**	-* *-	0.662	0.857	0.844	**0.017**	0.096	**0.010**
**β** **-sitosterol**	-* *-	0.267	0.074	**0.022**	0.884	0.439	0.805
**Campesterol**	-* *-	0.753	0.101	0.275	0.334	0.390	0.33
Δ**-5-avenasterol**	-* *-	0.194	0.462	0.066	0.338	0.208	0.342
**cholesterol**	-* *-	0.354	0.186	0.896	**0.044**	0.096	**0.049**
**total sterols**	-* *-	0.225	**0.04**	0.204	0.905	0.054	0.861
**(Uvaol+erythro)/sterol totaux**	-* *-	0.803	0.913	0.283	0.389	0.962	0.584

Bold values: each variable that has statistical significance for all tests was declared when *P values* are < 0.05; **∗**: each variable that has statistical significance for all tests was declared when *P values* are <0.05 and has biological relevance.

**Table 6:** Pearson's correlations of SAD.1 marker with fatty acid compositions and the PAL70 marker with olive oil parameters of the studied olive oil cultivars.

**Parameters**		**SAD.1**
	*r*	*p*

**Acidity**	0.684	**0.02**
**C16:0**	-0.083	0.809
**C16:1**	0.205	0.546
**C18:0**	0.644	**0.032**
**C18:1**	0.155	0.649
**C18:2**	-0.227	0.501
**C18:3**	-0.610	**0.046**
**C20:0**	0.350	0.291

		**PAL70**
	*r*	*p*

**Phenol**	-0.886	**<0.001**
**Oxidative Stability**	-0.884	**<0.001**
**Chlorophyll**	-0.814	**0.002**
**Sterol**	0.223	0.510

**Bold values**: each variable that has statistical significance was declared when *P* values are <0.05.

*P*: *P* value. *r*: correlation coefficient.

## Data Availability

All data generated or analyzed during this study are included in this published article.
